# Anæsthetics in Dental Practice

**Published:** 1888-10

**Authors:** J. C. Reeve

**Affiliations:** Dayton, Ohio


### THE DENTAL REGISTER.

VOL. XLII.] OCTOBER, 1888. [No. 10.

### COMMUNICATIONS.

### "Anæsthetics in Dental Practice."

BY J. C. REEVE, M.D., DAYTON, OHIO.

My attention having been directed to an article under the above heading published in the REGISTER for September, I take the liberty of making some comments upon the doctrines of that paper and of presenting some thoughts upon the subject at large.

The administration of anæsthetics is a matter of deepest interest to both the surgeon and the dentist, and nowhere do the two respective professions come in closer contact, or so intermingle as here. As I find myself unable to assent to much that is in that paper, and as I am decidedly opposed to the administration of such anæsthetics as chloroform and ether by dentists, I will commence by disclaiming any disposition to consider the paper or the subject in any other spirit than that of science. Farther, to clear away all suspicion of personal bias, I will say that there is no professional duty I perform so unwillingly as that of administering an anæsthetic for dental purposes, no fee that I consider so hardly earned as that which I receive for this service. At the same time I am frequently giving anæsthetics for general surgical purposes without hesitation and without undue anxiety. I am in the habit of advising those who come to me for this purpose to take nitrous oxide gas. But unfortunately there is, in the city in which I live, so far as I know, but a single dentist who administers this agent.

I waive all consideration of the point, as to it being far worse for the dentist than the surgeon to lose a patient under an anæsthetic. It is not a pleasant thing for either to go to bed on; but, happily I cannot speak from experience. I consider first the question: Are anæsthetics more dangerous in dental practice than in general surgery? The answer must be unqualifiedly in the affirmative. Without attempting to collect statistics take only those of the Royal Medico-Chirurgical Society and those of Sansom.* The one gives 8 cases of death under tooth-drawing out of 100 of all operations, and the other 12 out of 107. Here then is nearly 10 per cent. of all the deaths occurring in dental operations. But this statement alone gives no just idea of the relative mortality. This could only be accurately ascertained if the total number of administrations in all surgical operations was known. Certainly anæsthetics are administered for general surgical purposes hundreds of times for once in dental practice, and if so, then the relative number of deaths under tooth-drawing is enormously large.

The causes of the high rate of mortality during this particular operation are not far to seek. I do not believe that the entrance of blood into the air passage is very important. Several deaths, however, have been caused by an extracted tooth falling into the larynx, without doubt due to the position of the the patient. Anæsthetics should never be administered unless the patient be recumbent. This is not, however, in my opinion, a very potent factor and was fully considered in the paper. Another is the particular nerve involved in the dental operation, the acute pain caused by injuries to it and the powerful effect of sudden impressions upon its branches upon the great and vital processes of respiration and circulation. By sudden impressions upon this nerve more than any other, is that inhibition of the heart's action brought about which is sudden death. Far more important than all, however, is the fact that the induction of anæsthesia for tooth-drawing is likely to be incomplete, and will pretty certainly be so if the operator is also the administrator. Now it is a positive doctrine of the highest and latest authorities that such reflex actions as above given are increased under chloroform, that a state of

*On Chloroform, London, 1865, p. 69.

partial anæsthesia is therefore one of especial danger and especially so if the pain produced is at once sudden and sharp.* It is gratifying, therefore, to see that this source of danger is fully recognized by the author of the paper although it is not emphasized as it deserves to be. There is no more seductive procedure than to give a few whiffs of chloroform for the extraction of a tooth ; there is no more dangerous practice. If an anaesthetic is given at all, it should be given until the patient is "off." There is no plainer doctrine than this connected with the subject.

I must dissent *in toto* from the doctrine that a full dose of whisky before the administration of an anaesthetic secures safety. No one can have a higher respect than I have for the gentleman from whom the author of the paper quotes this practice, a practice which is by no means, however, confined to one person. But this doctrine of a stimulant insuring safety is a fallacy based upon the reasoning that "I have pursued this course so many hundreds of times, I never had an accident, therefore it is the thing to do." Now setting aside the miserable after effects of "a full dose of whisky," especially on delicate females, who are largely in the majority among dental patients, I can adduce from the record of deaths under chloroform many instances in which an alcoholic stimulant was given just before the fatal inhalation.

I regret to see the administration of bromide of ethyl recommended in the paper as I regret to see its use again advocated by men whose standing in surgery is high. I regret it, because I believe it to be a dangerous agent, and this belief is based upon: 1st, its bad record ; its rate of mortality is high when the number of times it has been administered is considered. 2nd, Its marked perturbative influence upon the heart's action. This effect was shown by three personal inhalations I made when this agent was first introduced.†

I looked with interest in the paper for such objections to the use of nitrous oxide as would justify dentists in resorting to the stronger anæsthetics. I could not find them. Those adduced

*See Lauder Brunton's Therapeutics, and Buxton on Anæsthetics, 1888.
†See "Two New Anæsthetics," Cin. Lancet and Clinic, April 10, 1880.

seem but trivial when the tremendous responsibility is considered which the dentist takes upon himself when he proceeds to administer chloroform or ether, when the awful calamity of a sudden death from these agents comes to mind. "All anæsthetics are dangerous."* It is the teaching of experience; it is a doctrine that no physiologist will doubt for a moment. But nitrous oxide has a record which is so enormously large, which presents so few instances of accident, that it seems to one who has given to this subject no small amount of study, most passing strange that a set of men who can use this agent in their practice, even with some discomfort and drawbacks, and perhaps considerable trouble, do not do so more generally.

I cannot close this article without a warning against the new-fangled anæsthetics which are being introduced for dental practice. I have investigated one and believe all to be what that one was—diluted chloroform. Now the free and equable dilution of chloroform with air is one of the best measures of safety, but it does not insure it. Death under chloroform has occurred, when the vapor was certainly, mechanically by an inhaler, kept down to what is generally the safe point. Death has occurred early in the process when but little had been poured from the bottle. It has occurred to those who had taken it safely before, even several times. The fact is chloroform is an uncertain remedy,† and as for ether the case referred to in the paper is enough for the experience of a life time.

Finally, the writer of the paper alludes to the appearance of a dentist before a jury investigating a case of death under an anaesthetic administered by him. How would he appear had he taken it upon himself to administer the agent and operate too, a thing which no surgeon ever does; or, if he was obliged to confess that what he administered was a secret preparation and its composition unknown to him!

*Buxton.

†See Report of the Anæsthetic Committee of the British Medical Association. in "British Medical Journal, 1879.

